# Pollen Foraging by Bumble Bee Queens During a Critical Nesting Period Revealed by DNA Metabarcoding

**DOI:** 10.1002/ece3.72733

**Published:** 2025-12-22

**Authors:** Kelsey Schoenemann, Haw Chuan Lim, Mia M. Keady, David E. Carr

**Affiliations:** ^1^ Department of Environmental Sciences University of Virginia Charlottesville Virginia USA; ^2^ Department of Biology George Mason University Manassas Virginia USA; ^3^ Department of Soil and Environmental Sciences University of Wisconsin‐Madison Madison Wisconsin USA

**Keywords:** *Bombus*, foraging, gyne, metabarcoding, pollen, queen

## Abstract

The nest‐founding stage represents an especially vulnerable period of the bumble bee (*Bombus*) life cycle, during which solitary queens must locate and collect sufficient foraging resources to sustain themselves and their brood. Yet, we lack contemporary information about floral foraging resources used by queens in early spring. Here, we use next‐generation sequencing to characterize the floral species used by queens for pollen provisions during early nest establishment. We collected pollen loads from over 100 wild bumble bee queens at working farms, rural and city parks, and nature preserves across the Piedmont region of Virginia, USA. Using metabarcoding of two universal DNA barcodes for plants, *ITS2* and *rbcL*, we determined the taxonomic composition of pollen used by queens. Pollen loads contained native and non‐native woody (e.g., *Cercis*: Fabaceae, *Prunus*: Rosaceae, *Salix*: Salicaceae), herbaceous (e.g., *Lamium*: Lamiaceae, *Viola*: Violaceae), and vine (e.g., *Lonicera*: Caprifoliaceae) taxa. The non‐native *Lamium* and *Elaeagnus* (Elaeagnaceae) most frequently hosted foraging queens, owing in part to their abundance across sites and the season. Pollen composition varied more over time than among bumble bee species or across sites, but land cover predicted a small amount of variation in pollen composition. Specifically, the percentage of crop land within 1 km increased the representation of *Lamium* in queen pollen loads, likely reflecting the abundance of the disturbance‐adapted flower in fallow cornfields. Finally, the pollen communities detected by *rbcL* were twice as diverse as those by *ITS2*, perhaps owing to the better taxonomic resolution afforded by the fast‐evolving *rbcL* marker. This study demonstrates that queens are flexible foragers and that among the most common *Bombus* species, plant phenology drives pollen use more than species identity. Further, this study highlights the importance of monitoring pollen diets to inform regional management strategies and considerations about metabarcoding techniques.

## Graphical abstract

During the nest‐founding stage of the bumble bee (Apidae: *Bombus*) life cycle, solitary queen bees gather floral pollen to feed their offspring in nascent colonies. Here, in the first study of queen pollen loads in the southeastern U.S., we use next‐generation sequencing to characterize the pollen collected by wild bumble bee queens across northern and central Virginia. We demonstrate that queens are flexible foragers and that (among the most common *Bombus* species) plant phenology drives pollen use more than bee species identity.

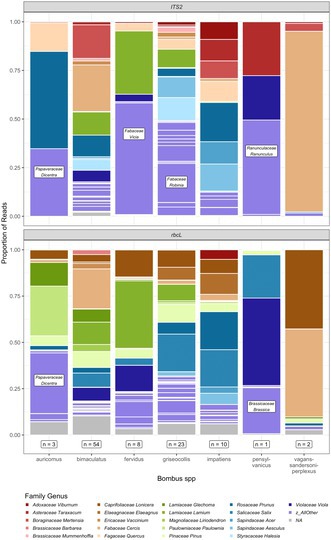

## 1 Introduction

The nest‐founding stage represents an especially vulnerable period of the bumble bee (*Bombus*) life cycle, requiring solitary queens to locate and acquire sufficient floral resources to feed herself as well as her brood. Like most bees, bumble bees depend on floral pollen and nectar for their entire diet. In particular, pollen provides the proteins, lipids, and micronutrients essential for larval growth and development (Roulston and Cane 2000); although its quantity and nutritional content vary among plant species (Tasei et al. 2008; Jeannerod et al. 2022). Once a colony is established, foraging bumble bees collect pollen in their corbicula (“pollen baskets”) to bring back to the colony and feed larvae. But bumble bee colonies do not persist from year to year. Instead, each year, queens emerge from hibernation and establish a new nest and perform all the necessary tasks (e.g., foraging, incubating, egg laying, cleaning) alone, until the first cohort of workers develops into adults. The pollen diet of queens before and after hibernation is critical for later colony fitness (Hollis Woodard et al. 2019; Watrous et al. 2019). Thus, abundant and high‐quality floral resources during the nest‐founding phase are likely key to queen survival and nest establishment.

However, many bumble bee species are in decline due to insufficient food resources. The decline of many *Bombus* species has been attributed to the loss of flower‐rich grasslands, for example, in the southern UK (Goulson et al. 2005) and northeast US (Richardson et al. 2019), as well as the simplification and intensification of cropland in the midwestern US (Hemberger et al. 2021). In each case, diverse flowering habitats have been lost due to the conversion of seminatural habitats to intensified agriculture and/or suburban development. In Virginia, land use change has taken the form of agricultural abandonment and forest conversion to accommodate urban expansion (Drummond and Loveland 2010; Sayler et al. 2016). In particular, between 1972 and 2000, the Piedmont region of the eastern US experienced the fastest urban development in the nation (Sayler et al. 2016), and about 71% of newly developed land replaced forest and 24% replaced agriculture. Between 1972 and 2000, forests shrank by 4.8% across the Piedmont; and in Virginia, farm numbers declined nearly 10% between 2012 and 2021 (Ellison et al. 2022). These changes are projected to continue into the future, yet we lack a modern baseline of *Bombus* queen foraging across these different landscapes. Understanding pollen resource use by queens across urban and rural landscapes would advance conservation of this group by identifying and conserving important food resources for bees (Williams and Osborne 2009; Dicks et al. 2013).

There is a paucity of queen foraging studies from the modern era, with no reports documented in the southeastern USA. Indeed, accounts of bumble bee queen foraging habits in early spring are rare and mostly date from before the turn of the century (Williamson 1911; Plath 1934; Fye and Medler 1954; Free 1968; Macior 1968, 1978, 1994; Thomson 1986; Olesen 1996), but see Lanterman et al. (2019), Anderson (2022), McCabe (2024). These field studies of queen pollen diets employ identification of plant species present in corbicular pollen samples (Macior 1968, 1978, 1994; Olesen 1996) and/or observations of foraging on flowers (all other citations). Historically, pollen samples have been identified by trained palynologists examining pollen grain morphology under a microscope.

Among reports from North America, researchers have documented queens foraging on native ephemerals (e.g., *Trillium*: Melanthiaceae, *Aquilegia*: Ranunculaceae), woody species (*Ribes*: Grossulariaceae, *Aesculus*: Sapindaceae, *Salix*: Salicaceae), herbaceous perennials (*Taraxacum*: Asteraceae, *Trifolium*: Fabaceae), introduced species (*Lonicera*: Caprifoliaceae, *Lamium*: Lamiaceae), and cultivated fruit crops (*Pyrus*: Rosaceae, *Malus*: Rosaceae) (Williamson 1911; Plath 1934; Fye and Medler 1954; Macior 1968, 1978, 1994; Thomson 1986; Lanterman et al. 2019; McCabe 2024). Between 50% and 60% of queen pollen loads contain a single pollen species (Macior 1968, 1978, 1994); however, queens do not appear to exhibit species‐specific or even caste‐specific tastes for pollen species (Teräs 1976; Ranta and Lundberg 1981). Instead, researchers note that differences in pollen collection or floral visitation are associated with the phenological succession and abundance of a limited suite of plants blooming during the period of queen emergence (Macior 1968, 1978, 1994; Ranta and Lundberg 1981; Lanterman et al. 2019).

The traditional method of identifying pollen load constituents is labor‐intensive and often yields low levels of taxonomic resolution (Rahl 2008). Meanwhile, observation of foraging behavior alone may fail to capture information about the pollen being collected if rare flowering plants or flowers in very tall trees are visited. Recent technical breakthroughs in next‐generation DNA sequencing have enabled the identification of plant species present in mixed pollen samples (reviewed in Bell et al. 2016, 2022). DNA metabarcoding is the high‐throughput sequencing and taxonomic assignment of universal DNA barcodes, which are present in all organisms' genomes and vary among species or genera. The number of sequence reads assigned to each taxon correlates semiquantitatively with DNA abundance in the sample extract, but study system, target region, and laboratory/bioinformatics methods can impact the strength of this correlation (Bell et al. 2016, 2022). Pollen DNA metabarcoding has already been used successfully to reveal patterns in the diets of wild, introduced, and historical populations of bumble bees (Bänsch et al. 2020; Simanonok et al. 2021; Ronca et al. 2023; Haque et al. 2024) without the time and labor costs of a trained palynologist or extensive field observations of foraging behavior.

Here, we report the first study of bumble bee queen pollen foraging across the Virginia landscape, using next‐generation DNA sequencing. We strategically selected sites representing a gradient in herbaceous, forested, and urban land covers and collected pollen samples from wild queens across these sites twice, 20 days apart, as well as continuously at one site for 3 months. We sequenced *ITS2* and *rbcL* barcodes and related patterns in pollen use across species and land cover and compared the data generated from the two barcodes. Specifically, we aimed to: (i) characterize the floral pollen sources most commonly used by queens; (ii) investigate differences in pollen species selection across queen species and in relation to land cover variation; and (iii) evaluate the consistency of pollen community data between two universal plant barcodes.

## 2 Methods

### 2.1 Study Area and Field Surveys

**FIGURE 1:**
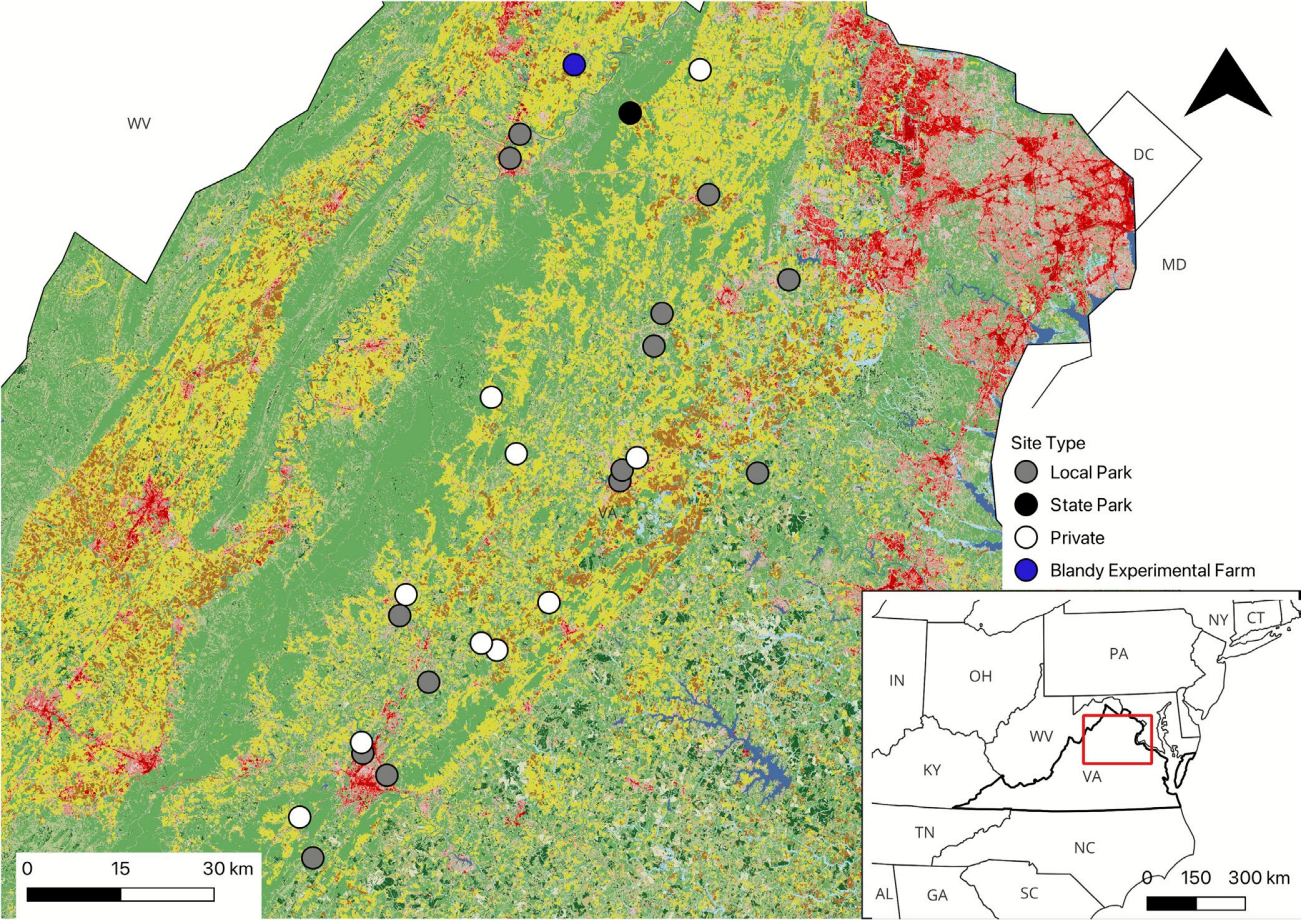
Map of 26 *Bombus* queen survey sites located in Virginia, USA, by ownership type. Basemap from the 2019 National Land Cover Dataset. Red box within inset represents the extent of the larger map.

To examine queen pollen collection in relation to land cover, from March 21 to April 30, 2022, teams of 1–4 surveyed wild queens at 15 public parks and 10 private properties in north and central Virginia, USA (Figure 1). To capture the most variation in land cover composition within the study region, we strategically selected 25 sites from a list of 85 candidate study sites, which included city parks, nature preserves, and working farms with more than two hectares of semi‐natural habitat. We performed principal component analysis (PCA) using the abundance of land cover categories within 1 km of each candidate (Homer et al. 2020), and selected sites that covered an even spread in multidimensional space (data not shown). The two primary axes described sites with more deciduous forest or more development, and more pasture/crop or more mixed forest/shrub.

Additionally, to examine patterns in pollen collection among *Bombus* species over the season at a single site, between March 30 and May 25, a single surveyor (DE Carr) searched for pollen‐bearing queens at Blandy Experimental Farm in Boyce, VA (hereafter “Blandy”; Figure 1). Blandy is an approximately 2.8 km^2^ biological research station, collocated with the State Arboretum of Virginia, and features over 5000 plant species in curation. The surrounding landscape is a mosaic of mixed deciduous forest patches and hedges, livestock pastures and fallow fields, croplands, and limited impervious or developed land.

All surveys took place on fair weather days (mean end temp = 18°C, range = 7°C–29°C, no continuous precipitation), between 9 am and 5 pm. At Blandy, the surveyor searched for queens on 26 days, about 2.2 days apart (range = 1–6 days), for an average of 107 min (range = 51–183 min) and covering an average of 4.1 km (range = 1.9–6.9 km) walking distance on each day. At all other sites, surveyors performed 60‐min timed surveys on two occasions about 22 days apart (range = 21–25 days). Surveyors attempted to capture every queen encountered.

To describe the suite of blooming plants available to foraging queens, we performed informal surveys of flowering species across the landscape sites (Lanterman et al. 2019). We did not record blooming plants at Blandy due to logistical constraints. At all other sites, we recorded all animal‐pollinated blooming plant species with more than two individual plants in bloom that we encountered during the survey. These resources were distributed in patches among tree, shrub, and herbaceous flower sources. We identified the plants to the lowest taxonomic level possible using dichotomous keys and descriptions from the Flora of Virginia (Weakley et al. 2012).

### 2.2 Queen Observation and Sample Collection

We identified the flower species on which queens were foraging at the time of capture, i.e., floral host, using the Flora of Virginia (Weakley et al. 2012). All queens were identified to species, except those of *B. sandersoni* , *B. perplexus* , or *B. vagans* (Milam et al. 2020). Instead, these queens were assigned to a species complex.

We removed pollen loads from queens with forceps, following immobilization by refrigeration. We cleaned forceps with alcohol wipes between samples. We stored pollen in microcentrifuge tubes at −20°C at the Blandy laboratory facilities until samples were processed at the George Mason University (GMU) Evolutionary Genomics lab (Manassas, VA). Bees were released after processing at the capture site.

### 2.3 DNA Metabarcoding

We extracted genomic DNA from pollen samples using the DNeasy Plant kit (Qiagen) following manufacturer guidelines. Negative extraction controls were included with each batch of 23 samples DNA extraction and processed through to sequencing. In brief, pollen samples were homogenized with ceramic beads (lysing matrix D, MP Biomedicals) in a FastPrep‐24 homogenizer (MP Biomedicals) and then chemically lysed with proprietary buffer. The plant DNA in solution was bound to a silica filter under high ionic (low pH) conditions and washed with buffers. Finally, the plant DNA was released from the silica filter in aqueous conditions.

We prepared indexed Illumina MiSeq libraries targeting two DNA barcodes, the internal transcribed spacer (hereafter, *ITS2*) and the ribulose‐1,5‐biphosphate carboxylase/oxygenase large subunit (hereafter, *rbcL*), using iTru “fusion” primers via a two‐step PCR (Glenn et al. 2019). These primers are universal plant barcode primers, *ITS2 S2F* and *ITS2 4R* from (White et al. 1990; Chen et al. 2010), *rbcL 2F* and *rbcLa R* from (Kress and Erickson 2007; Palmieri et al. 2009), with Illumina overhang adapter sequences appended, as in Glenn et al. (2019).

For the first step of the two‐step PCR, which we performed in duplicate, each 10 μL reaction consisted of 5 μL of KAPA HiFi HotStart ReadyMix (KAPA Biosystems, Boston, Massachusetts, USA), 0.3 μL of each of the forward and reverse primers of 10 μM concentration, 2.4 μL of PCR‐grade water, and 2 μL of DNA template. Reactions were performed in a thermocycler (Bio‐Rad T100) with the following conditions: initial denaturation at 95°C for 3 min, 29 cycles (for *ITS2*) or 32 cycles (for *rbcL*) of denaturation at 98°C for 20 s, annealing at 65°C for 15 s and elongation at 72°C for 15 s; followed by a final extension step at 72°C for 1 min. Negative controls with water instead of DNA template were included with each round of DNA amplification and processed through to sequencing. To confirm the presence of appropriately sized amplicons, 1 to 2 μL of each PCR product was visualized on an electrophoresis gel stained with GelRed (Biotium Inc., Fremont, CA) and illuminated by UV light. Successful duplicate reactions were combined. To purify the combined PCR products, we used Solid Phase Reversible Immobilization (SPRI) beads (Sera‐Mag SpeedBeads, Fisher Scientific) at a ratio of 1.5 bead solution volume to sample volume to isolate and discard fragments smaller than 100 bp. We eluted purified samples in 20 μL of molecular‐grade water.

For the second step of the two‐step PCR, which we performed in single reactions, each 15 μL reaction consisted of 7.5 μL of KAPA HiFi HotStart ReadyMix, 1.8 μL of each i5 and i7 primer of 5 μM concentration, 2.4 μL of purified PCR product, and 1.5 μL of molecular‐grade water. Thermal cycling conditions were as follows: initial denaturation at 98°C for 3 min, 9 cycles of denaturation at 98°C for 10 s, annealing at 62°C for 15 s, and elongation at 72°C for 30 s; followed by a final extension step at 72°C for 2 min. Negative controls with water instead of DNA template were included with each round of DNA amplification, but were discarded when gel visualization revealed that no product was present. Following another SPRI bead purification, we quantified the indexed products using the DeNovix dsDNA High Sensitivity fluorometric assays (DeNovix) and equimolar pooled individual samples into library pools. Samples or negative controls with concentrations less than 1 ng/μL were pooled at a standard volume that scaled with the total pool volume.

We further quantified the final *ITS2* and *rbcL* barcode libraries with High Sensitivity H1000 ScreenTape assay and pooled them together with libraries from four other projects (this project accounted for approximately 44% of the molecules sequenced). We pooled the *ITS2* and *rbcL* libraries with equal molarity relative to each other and conducted sequencing at GMU Evolutionary Genomics Lab with an Illumina MiSeq platform with a MiSeq v3 kit, generating 2 × 300 bp paired‐end reads. We applied a 12.7 pM dilution of final projects pool with a 5% PhiX spike‐in for quality control.

### 2.4 Land Cover Data

To describe the land cover composition surrounding survey sites and queen locations, we accessed the 2019 National Land Cover Dataset (NLCD), which provides nationwide 30 m resolution land cover data (Homer et al. 2020). Using QGIS version 3.34.11 (QGIS 2021), we extracted land cover within 1 km of survey site center points. In our study region, there were 15 land cover categories represented. After data extraction, we reclassified these 15 categories into water, developed, forest, herbaceous, and crop, based on their relative value for nesting and foraging opportunities for bees (Lanterman et al. 2019).

### 2.5 Analysis

All data analyses were conducted in RStudio with R version 4.4.2 (R Core Team; Vienna, Austria).

### 2.6 Bioinformatics

We used the R package “dada2” (version 1.32.0) for quality control, merging, and denoising of reads (Callahan et al. 2016), and the command line tool “cutadapt” (version 1.18) to remove primer and adaptor sequences from reads (Martin 2011). We excluded reads with ambiguous bases, shorter than 50 bp after trimming, or having more than 2 expected errors (except *ITS2* reverse reads could have 5 expected errors), and merged the forward and reverse reads with a minimum overlap of 11 bp for *rbcL* or 15 bp for *ITS2*. Then, we used dada2's denoising algorithm to infer amplicon sequence variants (ASVs) and generate a table of read numbers for each ASV. We removed chimeric sequences with dada2's “removeBimeraDenovo” function. Finally, we used the “decontam” package (version 1.26.0) to identify and remove contaminating DNA features with the “combined” method by assessing read frequency and prevalence relative to reads in extraction negatives (Davis et al. 2018). In total, we removed 1 sequence (of 345 total recovered) from the *ITS2* data and eight sequences (of 259 total recovered) from the *rbcL* data.

We used dada2's “assignTaxonomy” function to implement a naïve Bayesian classifier (Wang et al. 2007) with a k‐mer size of 8 and 100 bootstrap replicates to classify ASVs within a user‐supplied taxonomy of reference sequences. Only ASVs with more than 100 reads across all samples were assigned to taxa (Hammer et al. 2020, 2023). We used two publicly available reference databases compiled by scraping sequences of seed plants registered on NCBI: “PLANiTS” database for *ITS2* sequences (Banchi et al. 2020; last updated 2020), and the “rbcL” database for *rbcL* sequences (Bell, Fowler, et al. 2017; Bell, Loeffler, and Brosi 2017; last updated 2021). We restricted all analyses of pollen taxonomic assignments to the genus level to enable comparisons between barcode datasets; that is, while *ITS2* reads rarely assigned to species, both datasets usually assigned ASVs to the genus level (99% of *ITS2* reads, 93% of *rbcL*). None of the genera detected in our samples have homonyms in other taxonomic families. We created rarefaction curves with “rarecurve” function “vegan” (version 2.6) (Oksanen et al. 2025) to assess read depth and number of ASVs detected. We excluded samples with fewer than 1000 reads in total and samples with less than half of reads with assigned taxonomy.

### 2.7 Summarizing Pollen

Metabarcoding techniques generate compositional data that require appropriate transformations to meet assumptions of parametric community analyses. That is, the proportion of reads assigned to each taxon present in a sample will sum to one, which causes each taxon's proportional abundance to depend on the values of all other taxa. Log‐ratio transformations can increase the independence of these values (Tedersoo et al. 2022), while the centered log‐ratio (CLR) is commonly used in metabarcoding studies to meet the assumptions of community analysis (Pineda‐Mendoza et al. 2024; Wang et al. 2024; Mikhailov et al. 2025). Here, we used the “rcomp” and “clr” functions of the package “compositions” (version 2.0‐9) to transform the community data used for ordination (Van den Boogaart et al. 2025). First, we calculated a lower detection threshold from our dataset by dividing the minimum reads belonging to a single taxon by the maximum reads belonging to a single sample. Next, we included a dummy variable with a constant value 2% above the detection threshold, and we identified zeros that were “below detection threshold” with the “rcomp” function. These steps prevented zeros in the denominator of the CLR transformation (Mandal et al. 2015; Yerke et al. 2024). Finally, we used the “clr” function to apply the transformation to the dataset; and following the transformation, we removed the dummy variable. We employed this CLR‐transformed community dataset for nonmetric multidimensional scaling (NMDS) and redundancy analysis (RDA) as described in the following paragraph. For all other analyses of pollen composition, we used untransformed proportional read abundance.

We calculated genera diversity of pollen samples using the Shannon Index implemented with the “diversity” function of the “vegan” package (Oksanen et al. 2025). This metric reflects the relative richness and abundance of communities, returning larger values for communities that are taxa‐rich and evenly represented by each taxa. For this method, we excluded 28 genera appearing in only one sample.

We identified the most common genera across all queen pollen samples based on three metrics: (i) the number of samples in which each genus occurs, (ii) the average proportion of reads belonging to each genus in the samples in which it occurs, and (iii) the maximum proportion of reads belonging to each genus in the samples in which it occurs. We selected pollen genera that appeared in at least five samples, with an average of at least 2.5% of reads per sample and a maximum proportion of sample reads of at least 25%. Although proportional read abundance is not directly correlated with pollen mass (Arstingstall et al. 2023), plant genera present in more samples, and at larger proportions, are likely those more frequently visited by queens, as compared to other genera.

We classified species as “native” to our study region based on the Flora of Virginia (Weakley et al. 2012), which provides county‐level range maps with designations for native, introduced, and cultivated species. We identified genera containing invasive species according to the Virginia Department of Conservation and Recreation (Heffernan et al. 2024).

### 2.8 Comparing Across Barcodes

To assess the impact of barcode selection on pollen community detection, we compared the taxa identified in the *ITS2* and *rbcL* reference databases with those captured by each barcode using three distinct analytical methods. First, we assessed the overlap in taxonomic records between the *ITS2* and *rbcL* databases, as well as their overlap with the full set of genera detected in the samples. We also assessed the overlap among taxa detected in *ITS2* samples, *rbcL* samples, and the list of the most common genera across all samples.

Second, we compared Shannon diversity indices among *ITS2* and *rbcL* samples using nonparametric analysis of variance, the Kruskal–Wallis rank test, with the “kruskal.test” function in the package “stats” (version 4.4.2).

Third, to visualize how pollen composition differed among barcodes, we performed nonmetric multidimensional scaling using the “metaMDS” function from the package “vegan” (Oksanen et al. 2025) using the Euclidean dissimilarity matrix with CLR‐transformed proportions and two axes (*k* = 2) (Greenacre 2021). Before analysis, we excluded 28 genera appearing in only one sample (Cao et al. 2001). The best solution for NMDS ordination of all community data from both barcodes was not repeated and stress was high, so we also performed NDMS excluding poorly fit samples from the first NMDS plot or excluding samples without both barcodes. To test the effect of barcode identity on variation in community composition, we performed permutational multivariate analyses (PERMANOVA) with the “adonis2” function in the “vegan” package. To verify homogeneity of group dispersions, we used the functions “betadisper” to calculate the distances to the group centroids and “permutest” to implement ANOVA‐like permutation tests for homogeneity of multivariate dispersions (Shell and Rehan 2022).

### 2.9 Comparing Across *Bombus* Species

Sample sizes permitted statistical comparisons of pollen loads from *B. impatiens* , *B. griseocollis* , and *B. bimaculatus* . We compared average Shannon diversity across these three bumble bee species using a Kruskal–Wallis rank test. To visualize and test differences in pollen composition among these bumble bee species, we employed the same NMDS ordination and PERMANOVA with dispersion testing as described earlier.

### 2.10 Comparing Across Land Cover

To assess differences in Shannon diversity across land cover contexts, we fit a mixed effects linear model using the “lme” function of the “nlme” package (version 3.1‐167) (Pinheiro et al. 2025), excluding samples from the Blandy field site due to uneven sample size. In this model, we included four land cover variables (i.e., forest, herb, developed, crop) as explanatory variables, survey date, and sample collection time as covariates, and site as a random effect. Before analysis, we scaled and centered the explanatory and control variables. We performed a conditional *t*‐test testing the marginal significance of each fixed effect coefficient with the other fixed effects in the model.

Finally, to visualize and test the associations of pollen load composition across land cover contexts, we used redundancy analysis (RDA) with forward variable selection (Blanchet et al. 2008; Legendre et al. 2011). RDA is an extension of multiple regression, modeling the effect of an explanatory matrix on a response matrix (rather than a response variable). We modeled the CLR‐transformed read proportions (Greenacre 2021) with scaled and centered explanatory variables. Forward variable selection for the explanatory matrix follows a two‐step procedure: first testing the significance of a global model with all variables, and if the model is significant, proceeding with variable selection and stopping when the *p* value of the variable or adjusted *R* ^2^ is above a fixed threshold. Before analysis, we excluded 28 genera appearing in only one sample (Cao et al. 2001). We used the “ordiR2step” function from the “vegan” package to perform a forward selection process, adding explanatory variables to an initial null model from a set of candidates to maximize adjusted *R* ^2^ (Oksanen et al. 2025). The RDA global model included as predictors: percent cover of forest, herbaceous vegetation, developed land, and cropland within a 1 km radius, along with barcode, survey date, sample collection time, and latitude. For this analysis, we excluded data from Blandy samples due to uneven sample size. We included latitude to capture variation among pollen samples due to site identity.

## 3 Results

**FIGURE 2:**
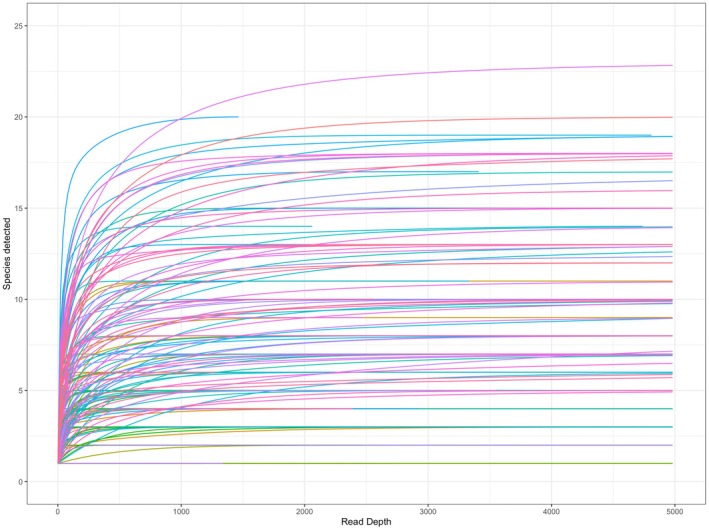
Accumulation curves for plant species detected in 101 queen pollen samples represented by one or two universal barcodes (*n* = 160 barcode‐samples). The *x*‐axis is truncated at 5000 reads to increase legibility.

We collected a total of 111 pollen loads from queens representing six species and one species complex (Table 1). After metabarcoding data quality control, we had data from 101 samples and 1,174,176 reads. Just over half of samples (59 of 101) were successfully amplified with both barcodes, while 32 samples were amplified with *rbcL* only and 10 were amplified with *ITS2* only. The average number of reads per barcode‐sample was 4652 reads for *ITS2* (range = 1062–16,430; SD = 3680), and 9376 reads for *rbcL* (range = 1161–22,161; SD = 5484). Sequence reads of both barcodes were almost always assigned to the family (99.6% of *ITS2*, 98.5% of *rbcL*) and genus level (99.3% of *ITS2* reads, 93.6% of *rbcL*); but while *rbcL* usually assigned to species (76.6%), *ITS2* reads were assigned to species less often (38.1%). Rarefaction shows that we achieved sufficient read depth to characterize the diversity of pollen in each sample (Figure 2).

**TABLE 1:** Summaries of sample size of pollen loads collected from *Bombus* queens.

Count of pollen samples across species and barcode				
*Bombus* species	ITS2 only	rbcL only	Both	Total
*Auricomus*	0	1	2	3
*Bimaculatus*	6	21	27	54
*Fervidus*	0	5	3	8
*Griseocollis*	4	4	15	23
*Impatiens*	0	0	10	10
*Pensylvanicus*	0	0	1	1
*Vagans‐sandersoni‐Perplexus*	0	1	1	2
Total	10	32	59	101

Count of pollen samples across sites				
Site	March	April	May	Total
All other	2	48	0	50
Blandy experimental farm	0	32	19	51
Total	2	80	19	101

*Note:* The summary across *Bombus* species reports the number of samples with data from *ITS2*, *rbcL*, or both barcodes and totals. The summary across survey site reports the number of samples from each survey month and totals.

**FIGURE 3:**
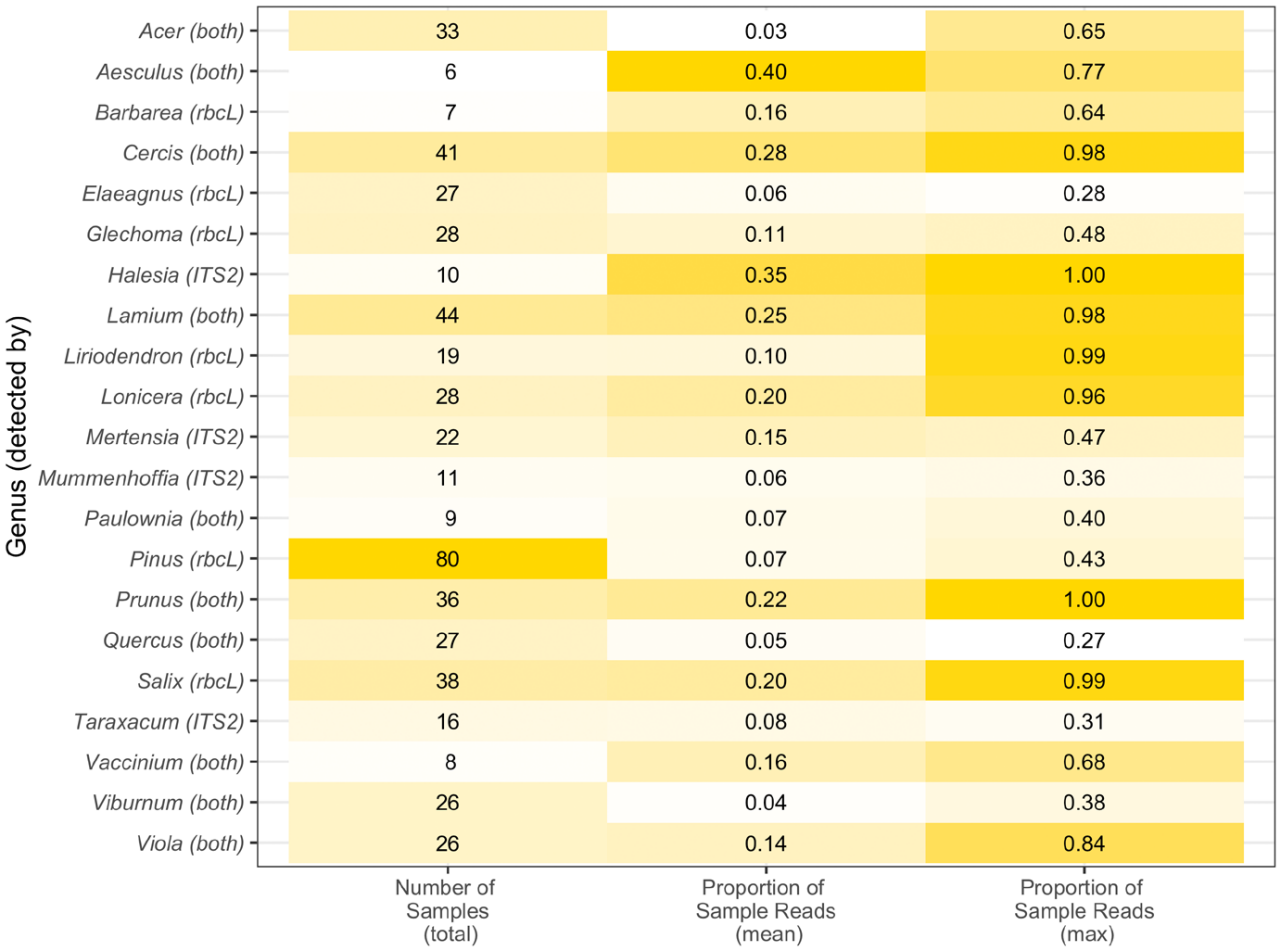
Occurrence of the most common plant genera detected in queen pollen loads with *ITS2*, *rbcL*, or both barcodes, in terms of genera prevalence among samples (number of samples), the average coverage of each genus within samples (proportion of reads), and the maximum coverage of each genus within samples (max proportion of reads), sorted alphabetically. Color intensity corresponds to the value of each metric. To be included, pollen genera must appear in at least five samples, accounting for at least 2.5% of sample reads on average, and with a maximum proportion of sample reads of at least 25%.

**FIGURE 4:**
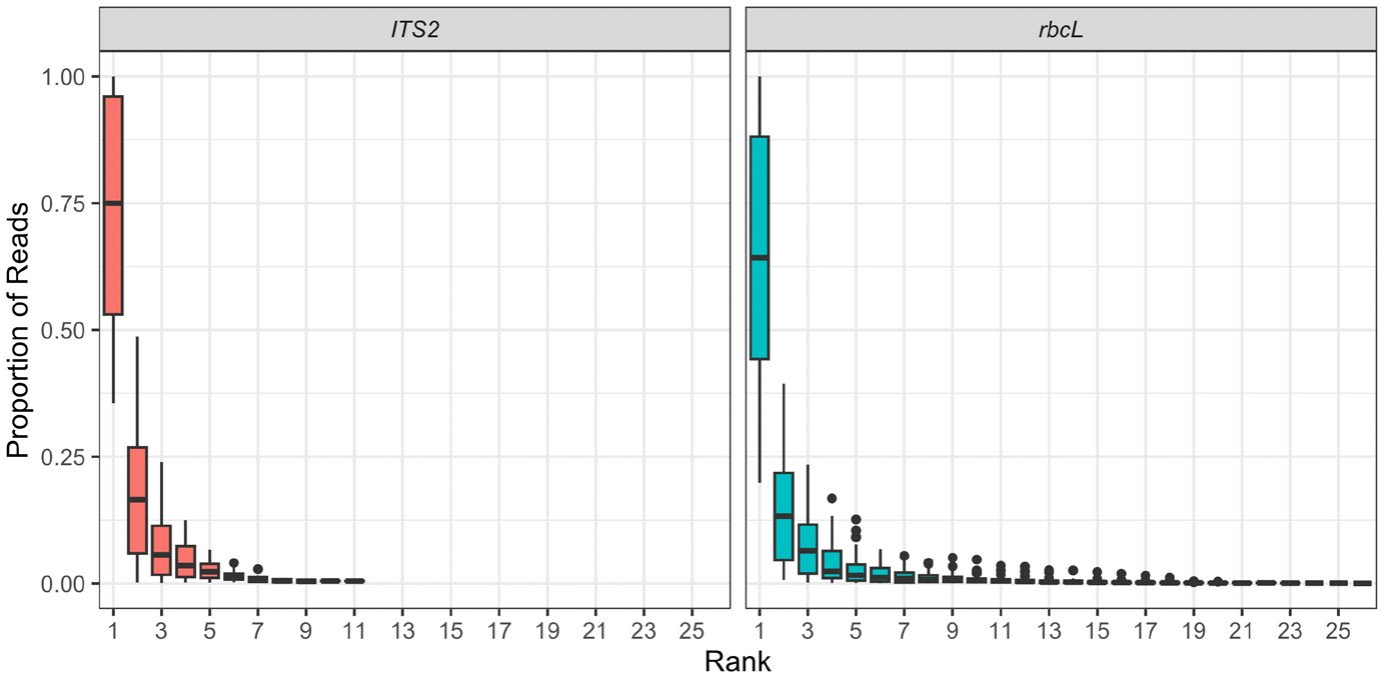
Rank abundance distribution of plant genera detected in 101 queen pollen loads with *ITS2* (left) and *rbcL* (right) barcodes.

A total of 95 genera were detected in pollen samples, but most data (83% of assigned reads) belonged to one of 21 genera (Figure 3). Each sample contained an average of 10.5 genera (4.0 for *ITS2* samples and 9.8 for *rbcL* samples) (*ITS2* range = 1–10, *rbcL* range = 1–21). However, most reads within a pollen sample belonged to three or fewer dominant plant genera (Figure 4).

### 3.1 The Most Common Genera

The most common genera included ephemerals (*Mertensia*: Boraginaceae, *Viola*: Violaceae), shrubs (*Cercis*: Fabaceae, *Halesia*: Styracaceae, *Vaccinium*: Ericaceae), trees (*Prunus*: Rosaceae, *Salix*, *Quercus*: Fabaceae), vines (*Lonicera*), and herbs (*Taraxacum*), including introduced species (*Elaeagnus*: Elaeagnaceae, *Lamium*) (Table 2). Seven genera detected in pollen samples contain no plant species native to Virginia, and three of those genera contain one or more invasive species (Table 2). Across barcodes, each genus was represented by fewer than two species on average (range = 1–4). However, because *rbcL* samples were assigned to species more often than *ITS2*, estimates of species richness may be biased in favor of *rbcL*‐detected taxa.

**TABLE 2:** Summary of sample read proportions and native/invasive status for the most common pollen genera detected in pollen samples.

Family	Genus	Detected by	Number of samples	Proportion of reads				Contains invasive species	Contains native species
				Mean	Min	Max	SD		
Aceraceae	*Acer*	Both	33	0.03	0	0.65	0.11	Y	
Sapindaceae	*Aesculus*	Both	6	0.4	0.04	0.77	0.29		
Brassicaceae	*Barbarea*	rbcL	7	0.16	0	0.64	0.24		N
Fabaceae	*Cercis*	Both	41	0.28	0	0.98	0.38		
Elaeagnaceae	*Elaeagnus*	rbcL	27	0.06	0	0.28	0.08	Y	N
Lamiaceae	*Glechoma*	rbcL	28	0.11	0	0.48	0.14	Y	N
Styracaceae	*Halesia*	ITS2	10	0.35	0	1	0.43		
Lamiaceae	*Lamium*	Both	44	0.25	0	0.98	0.33		N
Magnoliaceae	*Liriodendron*	rbcL	19	0.1	0	0.99	0.25		
Caprifoliaceae	*Lonicera*	rbcL	28	0.2	0	0.96	0.29	Y	
Boraginaceae	*Mertensia*	ITS2	22	0.15	0	0.47	0.18		
Brassicaceae	*Mummenhoffia*	ITS2	11	0.06	0	0.36	0.11		N
Paulowniaceae	*Paulownia*	Both	9	0.07	0	0.4	0.13	Y	N
Pinaceae	*Pinus*	rbcL	80	0.07	0	0.43	0.09		
Rosaceae	*Prunus*	Both	36	0.22	0	1	0.36		
Fagaceae	*Quercus*	Both	27	0.05	0	0.27	0.08		
Salicaceae	*Salix*	rbcL	38	0.2	0	0.99	0.32		
Asteraceae	*Taraxacum*	ITS2	16	0.08	0	0.31	0.11		N
Ericaceae	*Vaccinium*	Both	8	0.16	0	0.68	0.25		
Caprifoliaceae	*Viburnum*	Both	26	0.04	0	0.38	0.1	Y	
Violaceae	*Viola*	Both	26	0.14	0	0.84	0.26		

*Note:* Invasive species status from the Virginia Invasive Plant Species List and native status from the Flora of Virginia.

**FIGURE 5:**
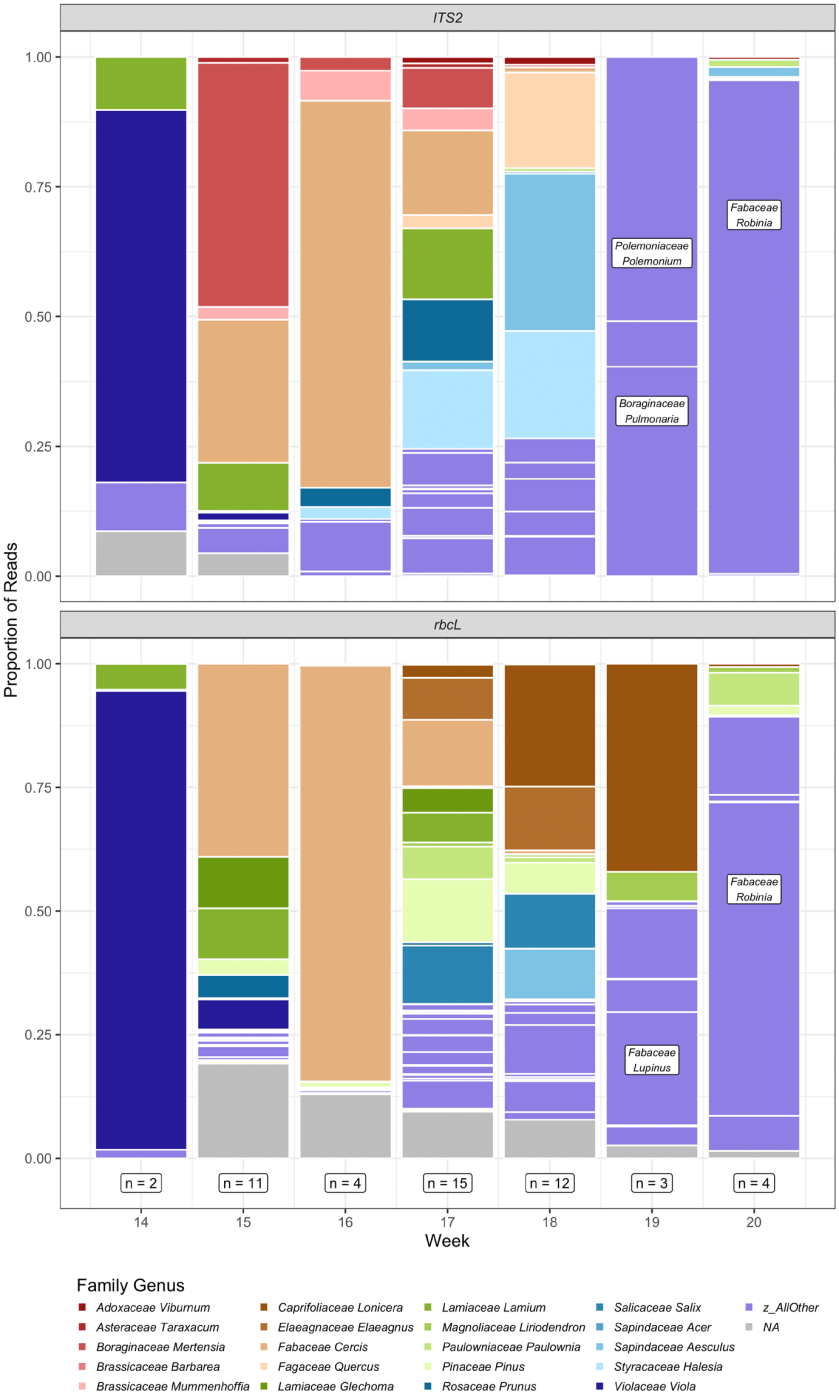
Proportion of reads assigned to plant genera in pollen samples collected from queens over time at Blandy Experimental Farm, using *ITS2* (top) and *rbcL* (bottom) barcodes. Numbered weeks start on: March 28, April 4, April 11, April 18, April 25, May 2, May 9, and May 16. Sample size for each week appears below the bars. The most common plant genera and “all other” genera are colored, while reads unassigned to genus are gray. Large proportions of “all other” genera are selectively labeled with text for clarity.

Although sample sizes were uneven, we observed temporal changes in queen pollen load composition at the one site with longitudinal data (Figure 5). Early samples were dominated by *Viola*, followed by a shift to *Cercis* in the subsequent week. *Cercis* declined in relative abundance in week 17, when we collected the most pollen samples. In the later weeks, *Lonicera*, *Halesia*, *Aesculus* were important, and the last samples collected contained *Lupinus* (Fabaceae) and *Robinia* (Fabaceae). Again, although proportional read abundance is not directly correlated with pollen mass (Arstingstall et al. 2023), plant genera present in more samples, and at larger proportions, are likely those more frequently visited by queens, as compared with other genera.

At the same time, across the landscape, the most commonly observed blooming plant genera (i.e., at > 31% of all site visits) were *Lamium*, *Viola*, *Taraxacum*, *Glechoma* (Lamiaceae), *Barbarea* (Brassicaceae), *Elaeagnus*, *Cornus* (Cornaceae), and *Cercis*. The species richness of blooming flowers noted in informal floral surveys increased over the sampling season, from an average of seven species per site in the first week to a peak of 12 species per site in the last week (Table 3). Queens were most frequently caught while foraging on flowers of *Elaeagnus umbellata* , *Glechoma hederacea* , or *Lamium purpureum* . These three species were used by 60% of queens carrying pollen in this study, and 73% of all foraging queens observed at the sites (Schoenemann 2025; *n* = 414 foraging bees). However, while *Glechoma* and *Elaeagnus* were very frequent floral hosts, they infrequently constituted much of the pollen load (i.e., accounting for > 25% of sample reads in 6 of 29 samples containing *Glechoma* and 4 of 32 samples containing *Elaeagnus*).

**TABLE 3:** Weekly floral species richness of study sites from informal (incidental) records of blooming plants encounters during *Bombus* queen surveys.

ISO week number	Week of	Floral species richness			Survey visits
		Mean	Min	Max	
12	3/22/22	7.33	5	10	9
13	3/30/22	7.17	5	11	6
14	4/4/22	8.75	3	13	8
15	4/12/22	9.38	5	14	8
16	4/22/22	8.75	5	13	8
17	4/29/22	11.6	5	15	8
18	4/30/22	12.5	8	17	2

### 3.2 Comparing Barcodes

**FIGURE 6:**
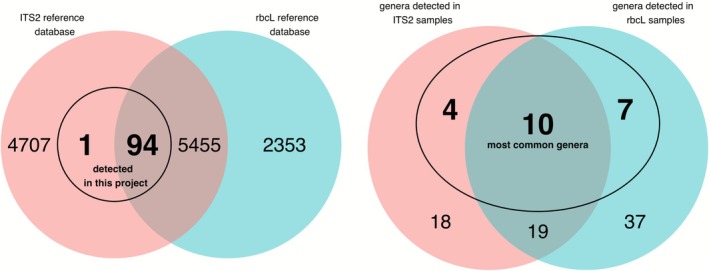
Left: Venn diagram illustrating the number of plant genera shared among the *ITS2* reference database (“PLANiTS” Banchi et al. 2020), the *rbcL* reference database (“rbcL” Bell, Fowler, et al. 2017; Bell, Loeffler, and Brosi 2017), and the 95 genera detected in queen pollen samples. Right: Venn diagram illustrating the number of genera shared among ITS2 pollen sample detections, rbcL pollen sample detections, and the most common pollen genera in the study.

**FIGURE 7:**
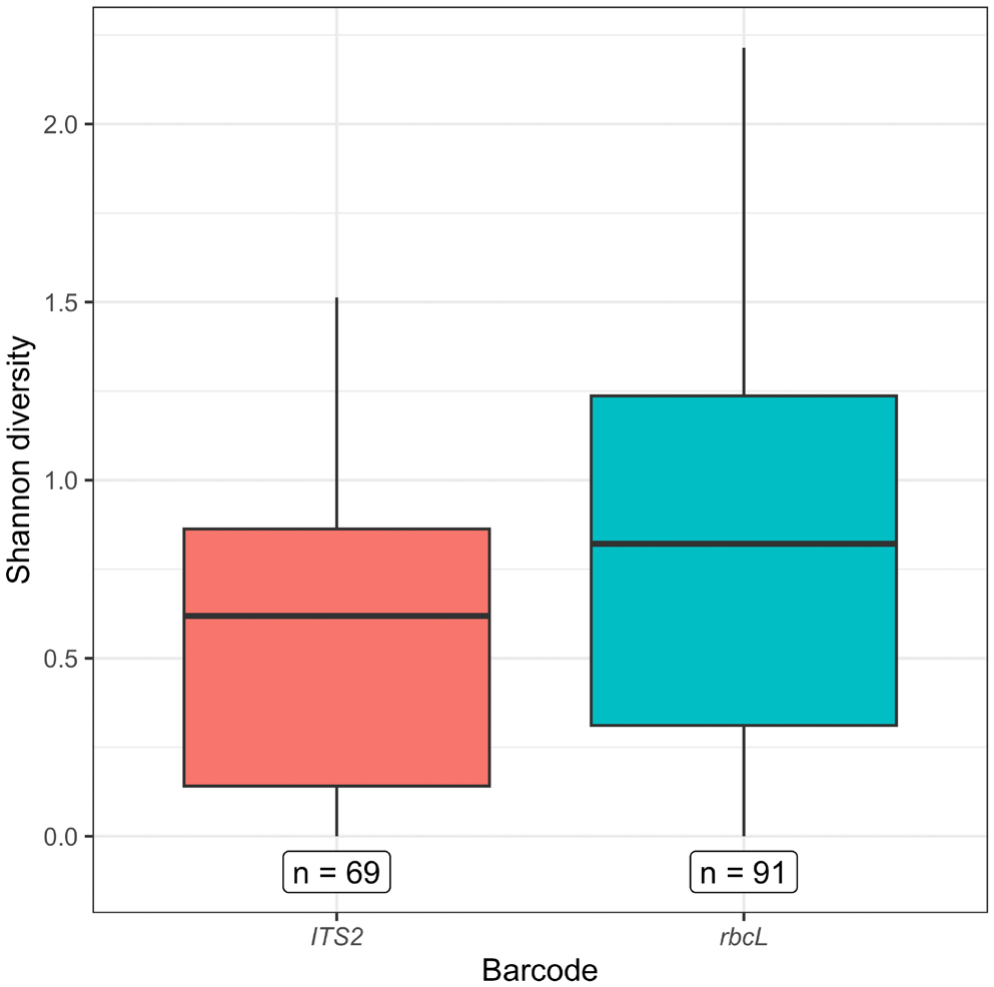
Boxplot of Shannon diversity index of queen pollen loads assessed with two barcodes, *ITS2* and *rbcL* (Kruskal–Wallis rank test; chi‐squared = 7.88, *p*‐value = 0.005).

**FIGURE 8:**
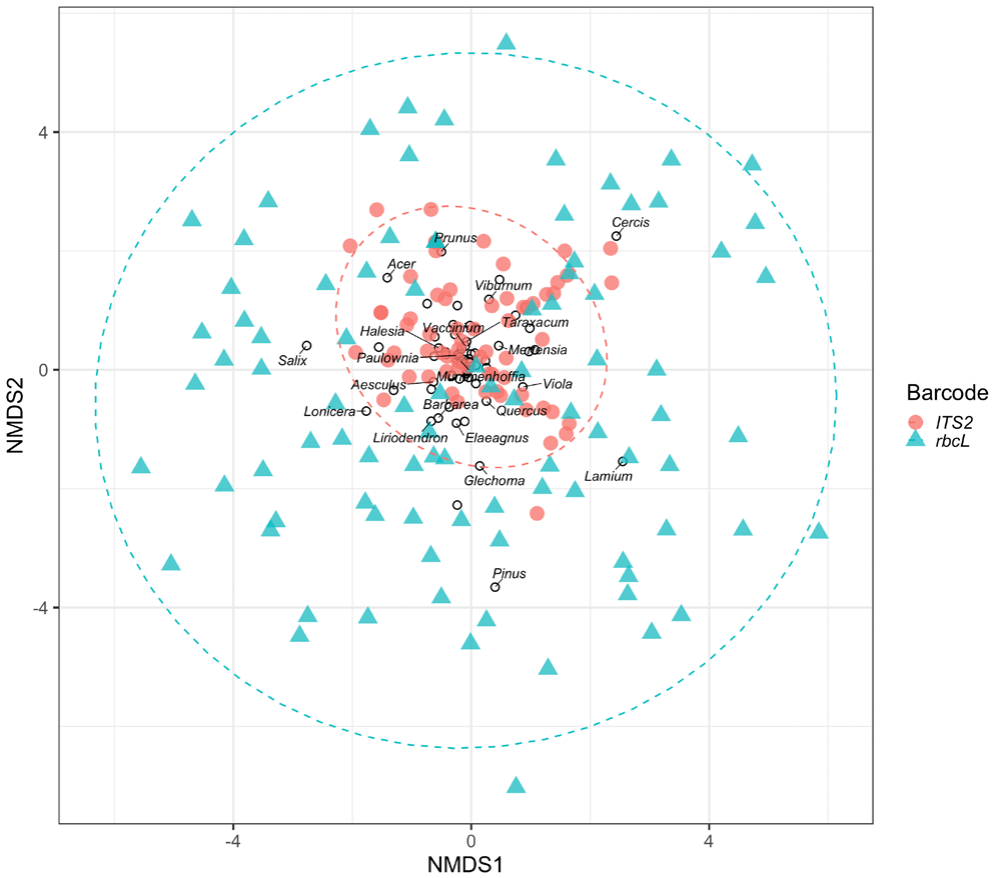
Non‐metric multidimensional scaling (NMDS) plot of pollen community composition comparing target barcode datasets (Stress = 0.26, *n* = 160 records from 101 bees). Genera comprising pollen samples are represented with empty circles.

All genera detected in this study, except one (*Mummenhoffia*: Brassicaceae), could be found in both barcode reference databases (Figure 6). However, a total of 65 genera were detected with only one barcode. There were 21 genera detected only with *ITS2* and 44 detected only with *rbcL* (Figure 6). Yet, we successfully amplified both barcodes in the majority of samples that contained barcode‐specific genera (53 of 83 samples with *rbcL*‐specific genera, 36 of 42 samples with *ITS2*‐specific genera), meaning that the detection of barcode‐specific genera in a sample was not due to missing data from the other barcode. On average, samples assessed with *ITS2* barcodes exhibited lower Shannon diversity indices than *rbcL* (Kruskal–Wallis rank test; Chi‐squared = 7.88, *p* value = 0.005, *n* = 160 barcode‐samples; Figure 7). Additionally, all NMDS plots showed pollen communities from *rbcL* overlapping with *ITS2* (Figure 8). However, all ordinations had high stress values (0.26–0.27) and no repeated best solutions, indicating that ordination poorly represents the data. Moreover, while we found community composition differed among barcodes (PERMANOVA; partial *R* ^2^ = 0.03, pseudo‐*F*_(1,158)_ = 5.45, *p* = 0.001), *rbcL* also exhibited significantly higher rates of dispersion (PERMDISP; *F*_(1,158)_ = 113, *p* = 0.001, 999 permutations). Therefore, we cannot evaluate whether the significant effect of barcode is due to differences in community dispersion or community location; but given the observed overlap in NMDS space, the former is more likely.

More than half of the most common genera were detected by a single barcode. Similarly, most families to which these genera belong were also barcode‐specific (2 of 3 *ITS2*‐specific genera, 6 of 8 *rbcL*‐specific genera). But three “most common” genera belonged to families that were detected by both barcodes. In these samples, the barcode sequences were assigned to the same family but different genera. For example, *Boraginaceae* reads were assigned to the *Mertensia* genus (a Virginia native plant) in *ITS2* samples, while in *rbcL* samples, reads of that family were assigned to *Cynoglossum* or, more often, unassigned to genus. Similarly, in *rbcL* samples, *Brassicaceae* reads were assigned to the *Brassica* genus, while in *ITS2* samples, reads of that family were assigned to *Cardamine*, *Mummenhoffia*, *Thlaspi*, or *Hesperis* genera.

### 3.3 Comparing Across *Bombus* Species

**FIGURE 9:**
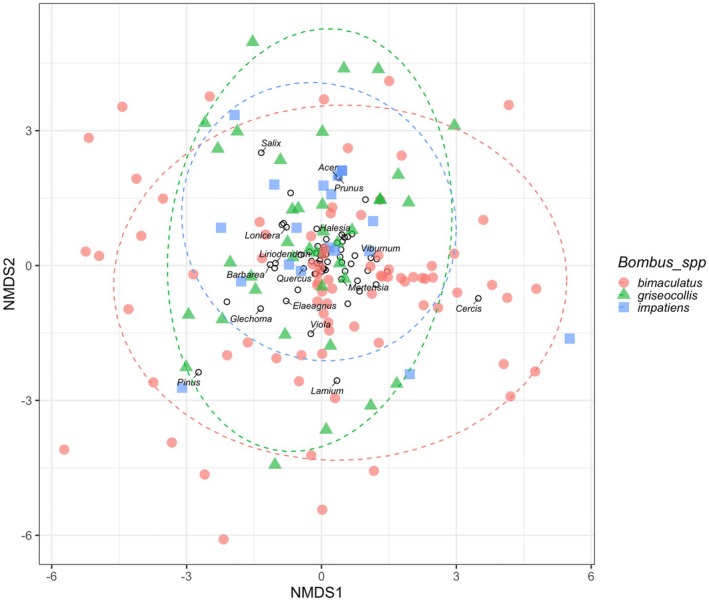
Non‐metric multidimensional scaling (NMDS) plot of community composition comparing pollen samples from three *Bombus* species (Stress = 0.26, *n* = 139 records from 87 bees), with data from *ITS2* and *rbcL* barcodes. Genera comprising pollen samples are represented with empty circles.

**FIGURE 10:**
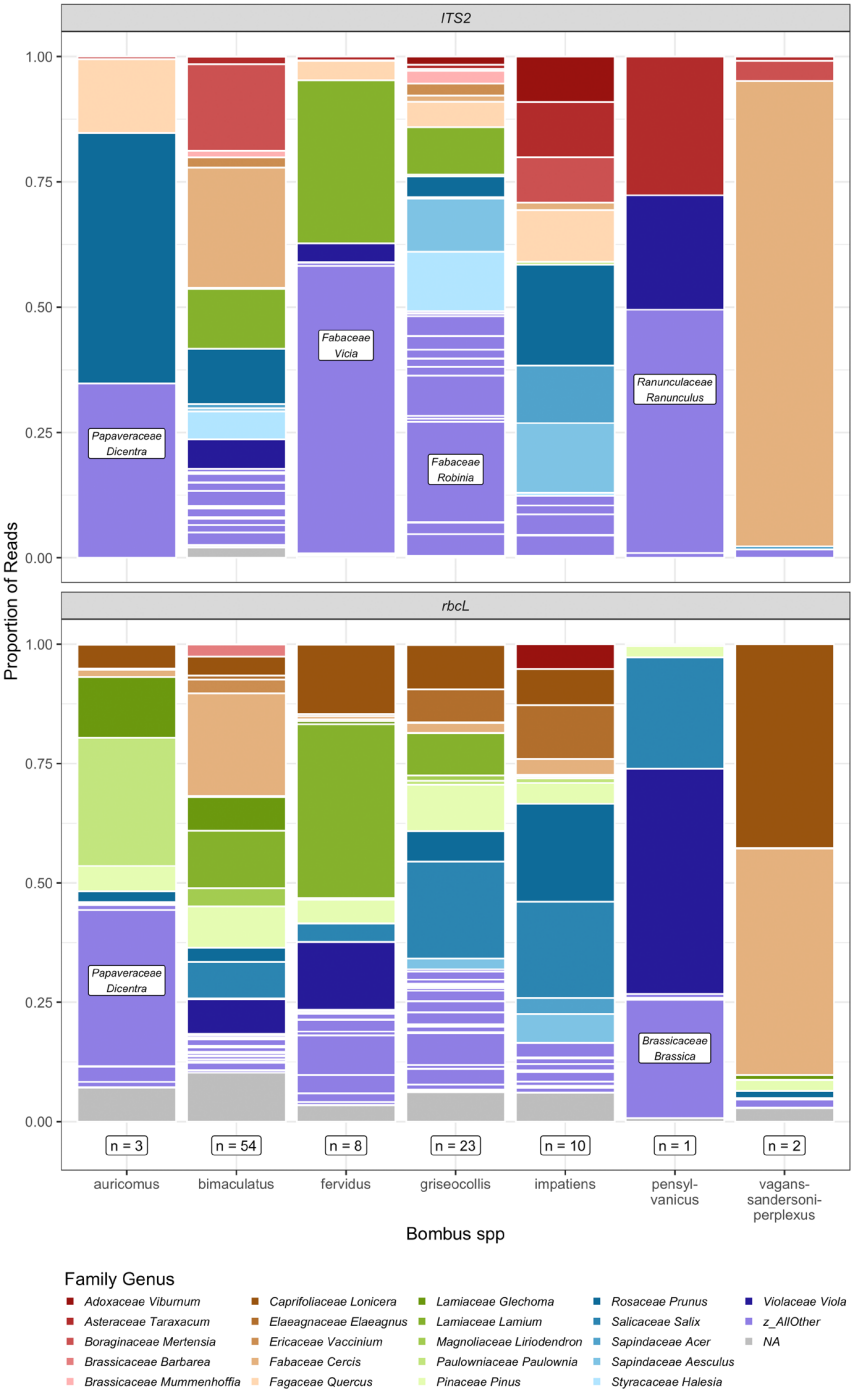
Proportion of reads assigned to plant genera in pollen samples collected from all *Bombus* species, using *ITS2* (top) and *rbcL* (bottom) barcodes. Sample size for each week appears below the bars. The most common plant genera and “all other” genera are colored, while reads unassigned to genus are gray. Large proportions of “all other” genera are selectively labeled with text for clarity.

The Shannon diversity index of pollen samples did not differ among the three most common bumble bee species (Kruskal–Wallis rank test; Chi‐squared = 0.399, *p* = 0.819, *n* = 87 pollen samples). In NMDS ordination (stress value = 0.26), pollen communities from the three common *Bombus* species overlapped (Figure 9), but PERMANOVA revealed significant differences in composition (PERMANOVA; partial *R* ^2^ = 0.04, pseudo‐*F*_(2,136)_ = 2.57, *p* = 0.001), which were not due to significantly different rates of dispersion (PERMDISP; *F*_(2,136)_ = 0.80, *p* = 0.47, 999 permutations). However, the ordination had a high stress value (0.25) and no repeated best solutions, indicating that pairwise sample distances should be interpreted with great caution. In general, we recovered greater proportions of reads for *Cercis*, *Viola*, and *Mertensia* from *B. bimaculatus* pollen samples, while *B. griseocollis* samples contained more *Robinia* and *Halesia*, and *B. impatiens* samples contained more *Prunus*, *Acer* (Aceraceae), *and Viburnum* (Adoxaceae) (Figure 10).

### 3.4 Comparing Across Land Cover

**FIGURE 11:**
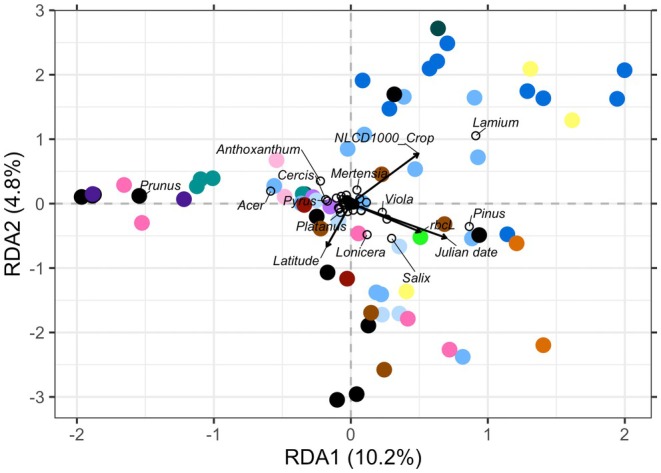
Redundancy analysis triplot of plant genera (empty circles) detected in pollen samples (colored points) collected from bumble bee queens, in relation to ordination axes based on land cover proportions within 1 km of survey site (adjusted *R* ^2^ = 0.16; *n* = 72 records from 50 bees at 15 sites). Colors represent survey site identity.

The average Shannon diversity index of pollen samples did not vary with any of the land cover classes but increased significantly with survey date (LMM, *t* value = 2.68, *p* value = 0.009; Table 4). In the final RDA model for land cover (adjusted *R* ^2^ = 0.16; *n* = 72 records from 50 bees at 15 sites), the percent of crop land cover was significantly associated with pollen community, as well as barcode, survey date, and site latitude (Table 5). In general, an increasing percentage of crop land within 1 km of survey sites increased the representation of *Lamium* pollen (Figure 11). Additionally, in a restricted analysis excluding samples represented by a single barcode, the effect of percent herbaceous and developed land was retained in the model (data not shown, adjusted *R* ^2^ = 0.31, *n* = 44 records of 22 bees at 11 sites). In this model, an increasing percentage of herbaceous land cover within 1 km increased somewhat the proportion of *Viola* and *Vicia* (Fabaceae) pollen in samples, while increasing developed land increased the representation of *Prunus* and *Acer* pollen in the samples. Forest cover explained the least variance of all variables.

**TABLE 4:** Summary output from linear mixed effects model describing the relationship between queen pollen load Shannon diversity and land cover proportions within 1 km of survey sites, survey date, and collection time.

	Value	SE	df	*t*	*p*
(Intercept)	0.703	0.073	55	9.644	0.000
Percent developed	0.213	0.961	10	0.221	0.830
Percent forest	0.279	0.915	10	0.304	0.767
Percent herbaceous	0.058	0.597	10	0.097	0.924
Percent crop	0.129	0.356	10	0.362	0.725
Date	0.177	0.061	55	2.917	0.005
Collection time	0.015	0.074	55	0.200	0.842

**TABLE 5:** Differences in queen pollen load composition from redundancy analysis with forward variable selection, describing the strength of relationships between pollen composition and explanatory variables.

	df	Variance	*F*	*p*
Date	1	2.076	5.919	0.001
Percent crop	1	1.942	5.536	0.001
Latitude	1	1.189	3.390	0.001
Barcode	1	1.117	3.183	0.001
Residual	67	23.5		

*Note:* Candidate variables included percent of forest, developed, herbaceous, and crop land covers within 1 km of survey sites, barcode dataset, survey date, collection time, and latitude. Adjusted *R* ^2^ = 0.16; *n* = 72 records from 50 bees from 15 sites.

## 4 Discussion

We found that queen pollen loads primarily consisted of one or two dominant plant genera, consistent with previous research, while simultaneously detecting a much greater taxonomic diversity than previously reported through traditional methods. We found 10 plant genera per pollen load on average, which is higher than the 1–4 species typically found in earlier microscopy‐based studies (Macior 1968, 1978, 1994; Olesen 1996). However, this increased diversity was driven primarily by minor constituents present in very low proportions—the two most abundant taxa still comprised more than 75% of the sequence data, corroborating earlier findings where queen pollen loads contained a single dominant constituent 50%–60% of the time (Macior 1968, 1978, 1994). This pattern reflects the foraging behavior of queens during nest establishment. Queens begin collecting pollen after they establish a nest and forage in smaller areas centered on the nest to do so (Cresswell 2000; Cresswell et al. 2000; Cavigliasso et al. 2020). In early spring, these floral resources are patchy and change over time. To maximize handling efficiency and take advantage of changing floral landscapes (Macior 1994; Grüter and Ratnieks 2011), bumble bees may specialize in collecting pollen from one or two flower species within a trip but forage from a diversity of plants across trips (Heinrich 1976, 1979; Vaudo et al. 2014; Somme et al. 2015). Although individual pollen loads contained mostly two dominant taxa, we also observed several minor pollen constituents in queen pollen, which may have been transferred to queen corbicular pollen loads incidentally during flower visits to drink nectar, assess pollen quality via ingestion (Mayberry et al. 2024), or from anemophilous pollen wafting in the air (e.g., *Pinus*: Pinaceae) (Pound et al. 2023). Thus, trace amounts of pollen from animal‐pollinated flowers may indicate visits by *Bombus* queens for purposes other than active pollen collection. It is important to note that DNA metabarcoding data are inherently compositional: increases in one taxon's relative abundance necessarily reduce the proportional representation of others, regardless of their absolute biomass. Therefore, below we focus on broad patterns in pollen composition rather than absolute abundances, and we interpret shifts in dominant taxa with appropriate caution (Deagle et al. 2019).

Pollen load composition changed over time at the one site with continuous sampling (i.e., Blandy Experimental Farm) (Figure 5), likely reflecting the bloom phenology of many species over time. At Blandy, *Cercis* comprised a large portion of pollen loads early in the season, but disappeared from pollen samples collected in the last weeks of the season. *Cercis* (assigned to *C. canadensis* , eastern redbud, with *rbcL* data) is a large shrub or small tree that blooms between March and May in Virginia and is commonly found in landscaping and forest edges (Weakley et al. 2012). As the season progressed, the number of plant taxa detected in pollen loads peaked and declined, reflecting total sample sizes. While *Cercis* is absent from samples beyond the fourth sampling week, only the final weeks of sampling feature pollen from *Lonicera* and *Robinia*. These genera represent vine and tree species that bloom from May to June and April to June, respectively (Weakley et al. 2012). Thus, the switch from *Cercis* to *Lonicera* and *Robinia* likely reflects bloom senescence, as well as the increase in availability of other floral pollen sources. Although we lack data from Blandy specifically, we noted the species richness of blooming flowers at other survey sites increased over the sampling season. Foraging bumble bee queens at Blandy therefore appeared to flexibly collect pollen from different plant taxa over time. We emphasize, however, that these temporal shifts represent changes in relative composition rather than absolute pollen quantities and should be interpreted within the constraints of compositional metabarcoding data.

Among the most common plant genera detected, we found herbaceous, woody, and vine taxa, while three genera consisted only of non‐native, invasive species (Table 2). These findings have implications for conservation plantings and invasive species management. First, our results demonstrate the importance of not just herbaceous wildflowers for bumble bee conservation, but also trees, shrubs, and vines. Queens have previously been documented using ephemerals and herbs (Macior 1978; Thomson 1986) as well as woody and vine species (Macior 1968) in the Midwest and northeast of North America. While conservation planting strategies for bees focus on herbaceous, insect‐pollinated species, woody and non‐animal pollinated species also offer important resources (Mola et al. 2021; Wood et al. 2022), and we show they are used by queens during nest‐establishment.

In this study, queens commonly visited non‐native and highly invasive species to collect pollen or nectar. We frequently observed queens on the invasive shrub, *Elaeagnus umbellata* (Heffernan et al. 2024) and the non‐native herb *Lamium* spp., which are considered occasionally invasive in West Virginia (WVDNR 2025). Queens that visit these species frequently enough to pollinate flowers will increase the plants' reproductive success, and thereby hamper control efforts. Indeed, queens likely pollinate *Elaeagnus* (Soley and Sipes 2021) and *Lamium* flowers (Sulborska et al. 2014; Lanterman et al. 2019). However, while *Elaeagnus* was a frequent floral host, it infrequently constituted much of the pollen load. Because read proportions are compositional, this low proportional representation does not necessarily indicate low absolute pollen collection, only that *Elaeagnus* contributed less relative to other concurrently collected taxa. Still, flower visitation without substantial pollen collection could indicate that the queens were nectar foraging or visiting pollen‐depleted flowers. *Elaeagnus* blooms produce a fragrant odor and abundant nectar (Hayes 1976; Soley and Sipes 2021). We are not able to determine, however, whether queens did not collect the pollen of these species because they were highly attractive plants quickly depleted of pollen, or because queens do not prefer the pollen these plants provide.

Additionally, about 10% of samples (*n* = 10) contained pollen from *Paulownia* (Paulowniaceae), with up to 50% of sample reads belonging to this genus. These reads were assigned to *P. tomentosa* , a widespread and highly invasive species in the study region (Heffernan et al. 2024). This tree can reach 10–25 m in height and produces large violet‐colored flowers in early spring; but, despite its prevalence, it escaped notice during informal floral surveys, and, furthermore no queens were captured while foraging in tree canopies. In this way, DNA metabarcoding helped detect a pollinator‐plant interaction that would have otherwise gone unnoticed (Arstingstall et al. 2021). Still, the proportional dominance of *Paulownia* in some samples should be interpreted cautiously, as compositional inflation can occur when other taxa are recovered in low quantities. Altogether, while non‐native species with earlier flowering times could bridge gaps in blooming resource availability, these resources could also represent a route of pesticide exposure (Fulton et al. 2019), even in semi‐natural wildlands (Long and Krupke 2016; Wagner et al. 2017). We suggest that herbicide use to control these invasive species should avoid the flowering period to reduce exposure of queens to their potential harmful effects (Weidenmüller et al. 2022; and reviewed in Zioga et al. 2020).

Among the bumble bee species with sufficient sample size for statistical comparison, *B. impatiens* , *B. griseocollis* , and *B. bimaculatus* , we found that the Shannon diversity of pollen loads did not differ, although we detected slight but statistically significant differences in composition. Samples largely clustered together in the ordination, which also had high stress. These results somewhat contrast with a recent field study of *Bombus* worker foraging in Ohio, which documented more pronounced differences in the floral diet of the same three species considered here (Lanterman Novotny et al. 2023). In general, our results reflect the different phenological traits and similar environmental conditions among queen species.

Slight differences in pollen composition among *Bombus* species reflect the nesting phenology of each species. Although pollen communities overlapped considerably among all three species in the ordination, *B. bimaculatus* utilized *Cercis* and *Mertensia* as pollen sources, while other species did not (Figure 10). This finding is partly explained by phenology. In the mid‐latitudes of central or eastern U.S., *B. bimaculatus* and *B. impatiens* queens begin collecting pollen 3–4 weeks earlier than *B. griseocollis* (Lanterman et al. 2019; Anderson 2022; Schoenemann 2025). *Cercis* and *Mertensia* were among the few plants blooming early in the season when *B. bimaculatus* forages for pollen, but by the time *B. griseocollis* begins foraging, a larger suite of foraging options was available. Indeed, *B. griseocollis* collected pollen from later‐blooming plants like *Robinia*, which *B. bimaculatus* did not. *B. impatiens* appears to have collected from the trees and shrubs *Prunus*, *Acer*, and *Viburnum* more than other *Bombus* species, but we had the fewest pollen samples from *B. impatiens* (*n* = 11), and post hoc rarefaction curves of pollen diversity among *Bombus* species indicated that the diversity of *B. impatiens* pollen loads had not reached a sufficient sample size to plateau. That is, a larger sample of *B. impatiens* queen pollen may reveal different patterns in the relative importance (frequency of use) of pollen genera.

Pollen communities were generally similar among *Bombus* species, with more pollen genera in common than not, and this finding could reflect similar floral choices available to queens across sites. All three of the common *Bombus* species were equally likely to be detected in each site and land cover context (Schoenemann 2025). Therefore, each species of *Bombus* foraged for pollen from the same potentially limited suite of floral resources in spring. Previous study within a *Bombus* species showed that workers of *B. vosnesenskii* collected pollen loads that were more similar within a field site than within a colony (Saifuddin and Jha 2014), suggesting foraging preferences are similar within a shared landscape. In that system, just three plant species contributed over 70% of the overall pollen diet of the *Bombus* colonies. Altogether, we did not find strong evidence of differing pollen foraging among the three most common *Bombus* species, and this may be due to the relatively limited diversity of flowering plants available in early spring.

Land cover differences among sites did not explain variation in Shannon diversity of queen pollen loads, but did explain some variation in pollen composition (Figure 10). These patterns should be interpreted as changes in the relative composition of pollen taxa rather than direct changes in absolute pollen collection, owing to the compositional nature of metabarcoding read data. Patterns of queen pollen composition across land cover reflect both floral availability and relative quality of species in a changing floral landscape. Queens are thought to disperse between 1 and 5 km after emerging from hibernation, but use smaller areas after nesting (Lepais et al. 2010; Makinson et al. 2019). We saw more *Lamium* in pollen loads from queens near large areas of cropland. This pattern likely reflects the fact that *Lamium* bloomed abundantly in these fields throughout the survey period. By area, most cropland in Virginia is corn, soybean, or wheat/barley (Ellison et al. 2022). Corn and soybean are typically planted in late April or by the end of May, and none of the fields visited in this study were planted with a winter cover crop. In these sites, frequent disturbance enables fast‐growing annual plants to colonize in periods between crop plantings. In the restricted model, excluding samples with data from a single barcode, we saw more *Prunus* and *Acer* pollen from queens in sites near developed land. In part, this finding could reflect that cherries and maples are common as street trees and in urban parks. Previous study has shown queens forage on *Prunus* pollen (Fye and Medler 1954; Macior 1968), while *Acer* and other trees have also been documented as pollen forage for bees (Wood et al. 2022; Filipiak 2024). We also saw more *Viola* and *Vicia* in pollen from queens near greater forest and herbaceous cover in the restricted model. These herbaceous flowers grow in fields and on forest edges. While *Vicia* has been noted as a queen forage plant before (Fye and Medler 1954; Lanterman et al. 2019; Anderson 2022; McCabe 2024), it appears not to be preferred (Jha et al. 2013; McCabe 2024). *Viola* has not been noted before.

Land use change affects plant communities and the spread of non‐native species, and thus impacts the floral resource availability for queens. In general, land use change in Virginia reflects rapid urban expansion that fuels agricultural abandonment and forest conversion (Drummond and Loveland 2010; Sayler et al. 2016). Conversion of agricultural and conversion or logging of forested areas likely decreases the availability of disturbance‐adapted *Lamium* available early in the year, as well as *Quercus*, *Acer*, and other woody tree pollen. At the same time, forest understories have become degraded in highly fragmented landscapes due to the combined effects of deer overabundance and invasive species, reducing the abundance of ephemeral plant species (Hanula et al. 2016; Gorchov et al. 2021). More fragmented forests are more easily colonized by non‐native vines like *Lonicera*, which have proliferated in the mid‐Atlantic and southeast, owing to their higher recruitment, growth rate, and tolerance to herbivory compared to native congeners (Ashton and Lerdau 2008; Schierenbeck et al. 2016; Matthews et al. 2016). Increased urban development means heavier management of open space and favoring of human‐selected species (like *Acer*, *Prunus*, and exotic herbaceous flowers). While we cannot assess whether the use of non‐native species is more common now, queens have been documented using non‐native species (e.g., *Lonicera*, *Pyrus*, and *Trifolium*) before, even 100 years ago (Williamson 1911; Fye and Medler 1954; Macior 1968). Altogether, given that these land use and vegetative community changes are projected to continue into the future, this study provides important baseline data on foraging patterns across the human‐dominated landscape of Virginia.

Our results showing five times more pollen taxa per sample (albeit in very small amounts) than previous research reflect the greater coverage and higher precision of metabarcoding compared to microscopy (Simanonok et al. 2022). While microscopy permits sampling only a tiny fraction of the pollen load (typically 200 grains total), metabarcoding techniques identify DNA from extracts of the entire pollen load, including minor constituents and environmental contaminants. Standard microscopy is sometimes unable to identify pollen grains beyond the level of family or genus, particularly when the taxa have similar morphologies (Rahl 2008). This “cryptic” diversity of pollen grains could be detected using metabarcoding, provided reference genomes are available to match detected pollen sequences to specific taxa. Comparative study finds that DNA sequencing can detect a greater number of taxa while depicting similar phenological patterns as microscopy (Smart et al. 2017). Nevertheless, in this study, because we restricted barcoding analyses to the genus level, the high pollen diversity observed is more related to greater sample coverage than revealing “cryptic” taxa missed by microscopy.

This study supports previous research that found both *ITS2* and *rbcL* regions were appropriate for taxonomic discrimination at the genus level, with high rates of generic assignments (Braukmann et al. 2017). However, we also detected major biases in how the primers amplify different plant genera. Overall, samples assessed with *rbcL* detected twice as many genera as *ITS2*, and each barcode detected taxa that the other did not. For instance, only *ITS2* detected *Halesia* and *Taraxacum*, while only *rbcL* detected *Salix* and *Lonicera*. In part, differences between the barcodes are related to lower genetic resolution in the *ITS2* sequences compared to *rbcL*. It is generally known that barcodes exhibit differential plant species discrimination and quantification (Kress and Erickson 2007; CBOL 2009; Costion et al. 2011; Bell, Fowler, et al. 2017; Bell et al. 2019; Arstingstall et al. 2023). Indeed, universal barcodes are harder to develop in plants than in other taxonomic kingdoms due to frequent paraphyly and weaker genetic differentiation among plant species (Fazekas et al. 2009).

The presence of *rbcL*, a plastid‐encoded gene, in paternal pollen grains may seem counterintuitive, given that angiosperms typically inherit plastid DNA (ptDNA) maternally. Indeed, about 80% of flowering plants appear to exhibit maternal plastid inheritance (Nagata et al. 1999; Sakamoto and Takami 2024). Yet, previous study shows plastid DNA can be successfully amplified from pollen grains (reviewed in Bell et al. 2016; Arstingstall et al. 2023). While the plastid genome is usually maternally inherited, the mechanisms for this process do not preclude the possibility of retrieving ptDNA from paternal pollen grains. Angiosperm pollen grains consist of vegetative cells with many ptDNA copies (Nagata et al. 1999; Twell 2010), and generative cells that may also retain plastids (Zhu et al. 1991; Chiu and Sears 1993; Reboudi and Zeyl 1994). The up‐ or downregulation of paternal ptDNA at these stages of development largely corresponds with species' paternal or maternal inheritance of the plastid genomes. Selective regulation of ptDNA during pollen development (e.g., mitotic divisions occurring before or after anther release, depending on the species) as well as after fertilization largely corresponds to species' paternal or maternal inheritance (Nagata et al. 1999; Sakamoto and Takami 2024). Differences among plant taxa in modes of inheritance and pollen development could explain why some species' ptDNA (and, by extension, the *rbcL* marker employed in this study) would be harder to detect in mixed pollen samples; namely, if they are of species with strict maternal inheritance and early pollen mitosis (i.e., before release from anther). However, limited literature exists to describe the mechanisms of plastid genome inheritance in most plant species, including the pollen genera that were only detected by the nuclear (*ITS2*) marker in this study. Among species with strictly maternal inheritance, paternal leakage occurs at low frequencies (Svab and Maliga 2007), with mounting evidence that suggests this pattern is common in plants (Wolfe and Randle 2004; Xu 2005; Azhagiri and Maliga 2007).

Other possible explanations for differences in pollen communities detected with *rbcL* and *ITS2* result from taxonomic variation and methodological biases. First, plant species vary in their pollen morphology, genome size, and marker copy number (Pacini and Hesse 2005; Michael 2014), which can impact DNA extraction efficiency (Pornon et al. 2016) and thus affect the total DNA template available for primer binding during amplification. We used DNA extracts without standardizing concentration, so extraction efficiency could explain some differences in barcode retrieval. Additionally, amplification biases can occur due to uneven template competition, primer‐template bias, and the generation of chimeric sequences (Porter and Hajibabaei 2018). We performed amplicon PCR in duplicate to mitigate PCR biases due to sample template stochasticity, but more study is needed. We might expect amplification biases because *ITS2* possesses highly conserved secondary structures, which can impact primer binding (Mai and Coleman 1997), while *rbcL* has no secondary structures. Additionally, our sequencing and bioinformatics approach was capable of sequencing barcodes up to 580 bp in length, but plant taxa whose barcode lengths exceed that limit will not be detected. *ITS2* can occur in the genome in multiple copies, and its length can vary considerably among taxa (Yao et al. 2010). Thus, it is possible that systematically higher variation in *ITS2* length explains the differences in barcode retrieval.

Additionally, although the two libraries were amplified and prepared separately and then equimolar pooled, we ultimately retrieved about half as many high‐quality reads for *ITS2* as *rbcL*. For PCR amplicon libraries like ours on the platform we used, the optimal cluster densities should be about 450 K/mm^2^, but we recorded densities in the range of 780 K/mm^2^. Lower nucleotide diversity and unbalanced libraries are more likely to be overclustered, which reduces total data output from the sequencing run. We found that the *ITS2* barcode data were less taxonomically diverse than *rbcL*. This finding contrasts multiple previous studies using both *rbcL* and *ITS2*, which reported higher taxonomic resolution with *ITS2* (Chen et al. 2010; Braukmann et al. 2017; Bell et al. 2021). It remains unclear whether the sequencing run was overclustered because *ITS2* was less diverse or whether *ITS2* was less diverse because we retrieved lower high‐quality data output due to overclustering.

Finally, bioinformatic methods and database representation also influence barcode performance (Bik 2021; Hleap et al. 2021). Close phylogenetic relatedness among genera in the same family, resulting in closely related taxa having identical DNA barcode markers, caused one barcode to be assigned to one genus, while another barcode of the same species was assigned to a different genus (Fazekas et al. 2009). *ITS2* has paralogues, related sequences in related taxa, that are under concerted evolution; thus, genes within a species may become more similar to other species in the same family. However, we found limited evidence of this artifact in our data. Altogether, of the 11 most common genera detected by a single barcode, only three showed evidence of a “barcode gap.” That is, we observed the different barcodes to assign reads to the same family but different genera in three cases. Moreover, the differences among barcodes do not appear to be related to reference database representation, as both databases contained reference sequences for the majority (> 98%) of taxa detected (Figure 6).

## 5 Conclusions

This study is the first account of queen corbicular pollen in southeastern USA, and the first using next‐generation sequencing. Conventional microscopy‐based pollen identification is labor‐intensive and requires taxonomic expertise, whereas metabarcoding enables high‐throughput, standardized processing of large sample numbers. Our results reveal that metabarcoding uncovered new insights into a poorly studied area of *Bombus* life history, highlighting queens' flexible foraging strategies and showing that plant phenology, more than queen species identity, shapes pollen use during nest founding. Further, this study highlights the importance of monitoring pollen diets to inform regional management strategies that promote plant species used by nest‐founding queens, and to tailor pesticide applications during this sensitive period. Metabarcoding was very effective at retrieving queen pollen and detected many genera not previously detected through other methods. Nevertheless, biases introduced by barcode selection were apparent, emphasizing the need for careful marker choice or the use of multiple DNA barcodes to more accurately assess pollen diversity and community composition. Finally, because our interpretations rely on compositional metabarcoding data, our conclusions emphasize shifts in relative pollen composition rather than absolute pollen intake, a distinction important for future studies aiming to link foraging behavior with nutritional ecology.

## Author Contributions

**David E. Carr:** conceptualization (equal), funding acquisition (equal), methodology (equal), resources (equal), writing – review and editing (supporting). **Kelsey Schoenemann:** conceptualization (equal), data curation (lead), formal analysis (lead), investigation (lead), methodology (supporting), writing – original draft (lead). **Haw Chuan Lim:** funding acquisition (equal), methodology (equal), resources (equal), writing – review and editing (supporting). **Mia M. Keady:** writing – review and editing (supporting).

## Funding

We thank the National Science Foundation NRT‐ROL (Award Number 2021791) for fellowship support; the 4‐VA partnership for funding to purchase laboratory supplies; and the Graduate School of Arts and Sciences student council for funding to purchase a new GPS. Collection from one Virginia State Park was permitted under the Virginia Department of Conservation and Recreation—Virginia State Parks (Research and collecting permit: ZZ‐RCP‐030722).

## Conflicts of Interest

The authors declare no conflicts of interest.

## Data Availability

The data (i.e., field observations, denoised and preprocessed molecular data) and R code that support the findings of this study are openly available in the “bumblebee_pollen” repository at https://github.com/EvoGenLab/bumblebee_pollen.
